# Glauber’s Salt Composites for HVAC Applications: A Study on the Use of the T-History Method with a Modified Data Evaluation Methodology

**DOI:** 10.3390/ma18132998

**Published:** 2025-06-24

**Authors:** Veeresh Ayyagari, Amir Shooshtari, Michael Ohadi

**Affiliations:** Department of Mechanical Engineering, A James Clark School of Engineering, University of Maryland, College Park, MD 20742, USA; veeresha@umd.edu (V.A.); ohadi@umd.edu (M.O.)

**Keywords:** Glauber’s salt, phase change material, T-history, melting point suppression

## Abstract

Phase change materials (PCMs) can be utilized in buildings for peak load shifting in air conditioning systems, and the use of salt hydrate-based PCMs can reduce the cost of thermal energy storage devices. Glauber’s salt is an economical salt hydrate PCM with a melting point of around 32 °C. However, the desired melting range typically falls between 18 and 22 °C for building air conditioning applications. Although many researchers have characterized Glauber’s salt and its composites with modified melting points, enthalpy–temperature curves for composites of Glauber’s salt and NaCl are unavailable. In this study, we report the melting and solidification enthalpy–temperature curves for two different composites of Glauber’s salt and NaCl with a melting point of 21 °C obtained by the T-history method. Both composites contain NaCl to suppress the melting point, borax to reduce supercooling, and sodium polyacrylate as a thickener to enhance cyclic stability. The first composite with 12 wt.% NaCl demonstrated 139 kJ·kg^−1^ of latent heat of fusion, and the second composite with 9 wt.% NaCl demonstrated 171 kJ·kg^−1^. Both the composites have high volumetric energy densities compared to their organic counterparts with similar melting points.

## 1. Introduction

Peak load shifting is a thermal management strategy that involves shifting the load during the peak period to the off-peak period. Thermal energy storage (TES) devices are a popular peak load-shifting solution for shifting the building’s thermal loads [1,2,3]. When applied to cooling load shifting, TES systems store the cold by running the air conditioners during off-peak hours, typically at night, and then they discharge the stored cold to the building during peak load hours. Through this peak load shifting, TES provides energy and monetary savings [4]. TES systems are more cost-effective than lithium-ion batteries for shifting thermal loads [5].

TES systems often employ phase change materials (PCMs) to store and release thermal energy during the phase change process. Ice is the most cost-effective PCM available on the market and has been implemented as a PCM in TES devices [6,7,8]. However, the ideal phase transition temperature of PCMs for building cooling applications for both residential and commercial buildings, such as data centers, is around 18–25 °C [9,10,11].

**Figure 1:**
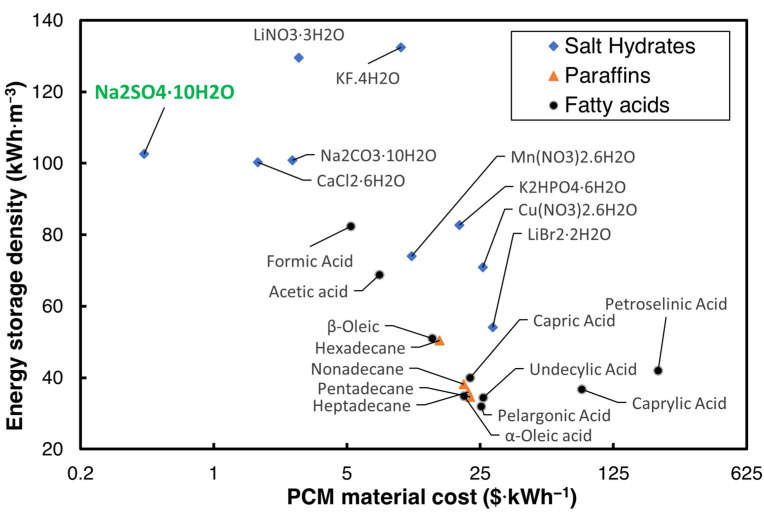
Energy storage density vs. cost of PCM [13].

The cost of the PCM plays a critical role in commercializing the TES technology, and many options of PCMs are available in the literature for HVAC applications, which have phase transition temperatures between 18 and 25 °C. The U.S. Department of Energy (DOE) has set a USD 15/kWh goal for the cost of the PCM with a volumetric energy storage capacity greater than 288 MJ·m^−3^ for use in buildings [12]. Hirschey et al. [13] reviewed the cost of several PCMs in building cooling and heating operating ranges. Of the plethora of PCMs reviewed, salt hydrates were identified as lower-cost PCMs compared to organic PCMs. The maximum cost of the PCMs provided by Hirschey et al. [13], and the volumetric energy storage density for three widely used categories of PCM for air conditioning applications—salt hydrates, paraffins, and fatty acids with a melting temperature $T_{m}$ ranging from 10 to 35 °C—are shown in Figure 1.

As can be seen in Figure 1, Na_2_SO_4_·10H_2_O, sodium sulfate decahydrate (SSD), also commonly known as Glauber’s salt, is one of the most inexpensive salt hydrates that can be used to achieve the DOE’s cost goal. While SSD is an economical PCM with a low cost and high energy storage density, it suffers from issues such as high supercooling, phase segregation, and non-optimal $T_{m}$ for HVAC applications.

Supercooling refers to the phenomenon where the PCM material remains in a liquid state below its nominal freezing point without undergoing solidification. This occurs when the material does not spontaneously nucleate and transition to the solid phase, even though it is below its freezing temperature. Supercooling can lead to delayed phase change behavior, impacting the reliability and efficiency of PCM-based thermal energy storage systems. Minimizing supercooling is essential to ensure the predictable and consistent performance of PCMs. Adding nucleating agents to the PCMs to suppress the degree of supercooling is a widely used method in the literature. Borax is the most popular nucleating agent, which, when added at 4 wt.%, reduces SSD’s degree of supercooling from 14 °C to 4 °C [14].

Phase segregation refers to the separation of different components within a PCM during melting or solidification, which can negatively impact the material’s thermal performance. For temperatures above the $T_{m}$ of SSD, the solubility of Na_2_SO_4_ in H_2_O is very low. Thus, SSD melts incongruently, and with repeated melting and solidification, the separation between the Na_2_SO_4_ and H_2_O phases increases. This phase segregation results in decreased contact between the phases, limiting the rehydration of Na_2_SO_4_ during solidification and thereby attenuating the energy storage capacity. The loss of energy storage density is further exacerbated by cycling between melting and solidification. As an example, SSD with borax added as a nucleating agent, without any further additives, loses 75% of its latent heat of fusion $H_{f}$ in 20–40 cycles [15].

Adding thickeners, mechanically stirring the PCM, and adding excess water [16] are a few of the proposed methods to overcome phase segregation. Microencapsulation of PCM is another method to overcome phase segregation and low thermal conductivity and to fine-tune the properties of the composites [17]. However, microencapsulation is an expensive process. Adding thickeners to PCM is a popular low-cost method to improve their handling and cyclic stability. Thickeners enhance viscosity and help maintain a uniform dispersion of components to prevent phase separation and leakage during storage and application. Thus, the thickening method was chosen as a method of enhancing cyclic stability in the current study.

SSD melts at approximately 32 °C and may not be suitable for building cooling applications. The properties of SSD could be tailored to suit the target application through additives. The $T_{m}$ of SSD can be adjusted by adding ionic salts such as sodium chloride (NaCl) or potassium chloride (KCl), which cause stretching in the bond between Na_2_SO_4_ and H_2_O molecules, leading to a lower $T_{m}$, referred to as a composite [18]. Alternatively, combining different PCMs with different melting points, such as sodium carbonate decahydrate (Na_2_CO_3_·10H_2_O) combined with SSD, can form a eutectic mixture with a $T_{m}$ lower than 32 °C [19]. Table 1 summarizes the reported studies on the melting properties of pure SSD, eutectics, and composites of SSD, where the $T_{m}$ of the SSD is modified.

Akamo et al. [19] tested the combination of SSD and Na_2_CO_3_·10H_2_O in multiple ratios and concluded that the 50 wt.% SSD–50 wt.% Na_2_CO_3_·10H_2_O eutectic of SSD provided the lowest $T_{m}$ of 25.18 °C with $H_{f}$ = 187.46 kJ·kg^−1^. Fang et al. [20] studied the eutectic mixture of SSD and Na_2_HPO_4_·12H_2_O and reported a $T_{m}$ = 25.86 °C with a $\;H_{f}$ of 210 kJ·kg^−1^ for a 20 wt.% SSD–80 wt.% Na_2_HPO_4_·12H_2_O. To reduce the supercooling, they added 2.5 wt.% Na_2_SiO_3_·9H_2_O, and to improve the cyclic stability, they added 30 wt.% SiO_2_. The composite demonstrated a $T_{m}$ = 25 °C and a $H_{f}$ of 143 kJ·kg^−1^. Hou et al. [21] prepared a composite PCM with SSD as a base PCM with a lowered $T_{m}$ using 5 wt.% KCl. They thickened the composite using 5 wt.% polyacrylamide (PAM) and added 3 wt.% expanded graphite to enhance its thermal conductivity. They reported a $H_{f}$ of 111 kJ·kg^−1^ with a $T_{m}$ = 23.6 °C for the composite. Jiang et al. [22] used a combination of 10 wt.% NaCl and 10 wt.% KCl for melting point suppression and carboxymethyl cellulose (CMC) as a thickener. The composite PCM achieved a $T_{m}$ = 13.4 °C and a $H_{f}$ = 128 kJ·kg^−1^.

Olson et al. [23] investigated the effect of adding NaCl to SSD on $T_{m}$ with CMC as a thickener. They reported that additions of up to 5% NaCl decreased the $T_{m}$ of the composite to about 25 °C. They also noted that further addition of NaCl did not have a significant effect on the melting point suppression. However, a comprehensive thermophysical characterization of the PCM was not carried out. Ryu et al. [24] investigated sodium polyacrylate (SPA) and CMC as thickeners with SSD, and experimentally concluded that SPA is a better thickener for high-hydration salts such as SSD, while CMC works better for low-hydration salts such as sodium acetate trihydrate (SAT). When using 3 wt.% SPA for thickening and 4 wt.% borax to promote nucleation in SSD, the authors observed that the $T_{m}$ of the composite PCM remained unchanged at 32 °C and reported a $H_{f}$ = 227 kJ·kg^−1^.

All of the SSD composite characterization studies in Table 1 used the digital scanning calorimetry (DSC) method for PCM thermophysical characterization. However, the DSC method has certain limitations when characterizing salt hydrates. Nucleation theory suggests that supercooling is a statistical process and that the magnitude of supercooling is high [22], while phase separation is suppressed [23,24] in smaller volumes. DSC employs small sample sizes ranging from 1 to 50 mg. Thus, using DSC could lead to discrepancies in characterizing thermophysical properties such as supercooling and cyclic stability, and the measurement results may not accurately represent the behavior of bulk PCM.

Zhang et al. [25] were the first to introduce the T-history method, which characterizes the PCM thermophysical properties by comparing the temperature–time trends of PCM and a reference material, by subjecting them to a sudden temperature change. It is as a cost-effective alternative to DSC for assessing bulk PCM properties. Unlike DSC, the T-history method accommodates larger sample sizes—up to 15,000 mg of PCM, which is 2000–3000 times larger than DSC, thereby solving the representative problem of bulk PCMs. Moreover, it is simple, affordable, and sustainable and can be assembled using basic laboratory equipment. Since salt hydrates generally suffer from significant supercooling and are prone to phase segregation, the T-history method is suitable for estimating their thermophysical properties [26,27]. Thus, the T-history method was chosen for the current study to characterize the SSD composites.

Since the introduction of the T-history method to characterize the PCMs by Zhang et al. [25], many researchers have proposed modifications to the test setup and advancements in data evaluation. Marin et al. [28] introduced an enhanced mathematical evaluation method, producing enthalpy–temperature curves crucial for TES design and validation, such as those that rely on enthalpy–porosity schemes [29,30,31,32]. Sandnes and Rekstad [33] proposed an alternative methodology to compute the enthalpy–temperature curves for PCMs that demonstrate significant supercooling. Kravvaritis [34] improved the accuracy by insulating test tubes to emphasize conduction as the primary thermal resistance path, reducing the Biot number (Bi). Despite these improvements, all methods are rooted in lumped capacitance principles, differing mainly in data analysis or experimental setup adjustments aimed at enhancing data reliability. D’Avignon et al. [35] compared the above-mentioned four T-history methodologies, advocating for Marin’s method [28] with specific modifications suited for salt hydrates with significant supercooling. The above-mentioned studies have their advantages and pitfalls, prompting the authors to introduce a data evaluation methodology that combines these methodologies, tailored explicitly for salt hydrates.

It is essential to use low-cost additives to keep the composite cost low, and NaCl and SPA are suitable, low-cost, and readily available options. However, the thermophysical properties of SSD combined with NaCl and SPA are unknown. Therefore, this study aims to characterize two SSD composites containing sodium chloride (NaCl) for its melting point suppression properties, in combination with sodium polyacrylate (SPA) to enhance cyclic stability as a thickener, and borax to reduce the supercooling degree as an additive.

Since the composites under study are impure, non-homogeneous, and demonstrate significant supercooling, DSC is not an appropriate method for their characterization. Therefore, the T-history method was chosen to characterize the composite PCMs in our study. Due to significant supercooling in the composites, a variant of the data evaluation method introduced by Sandnes and Rekstad [33] was implemented to obtain accurate enthalpy–temperature curves. The motivation for using this hybrid method is discussed in the upcoming section.

## 2. Materials and Methodology

### 2.1. Experimental Setup

We procured 99% pure anhydrous sodium sulfate (ThermoFisher Scientific, Waltham, MA, USA), anhydrous borax (Sigma Aldrich, St. Louis, MO, USA), SPA (Sigma Aldrich), NaCl (Alfa Aesar, Haverhill, MA, USA), and n-Hexadecane (Alfa Aesar) from commercial suppliers. The borosilicate test tubes (Fisher Scientific, Hampton, NH, USA) were used to hold the reference materials and PCMs.

Reduced fine-tip-diameter K-type thermocouples (Omega Engineering, Norwalk, CT, USA) were used to measure reference and PCM temperatures for a faster thermal response. The ambient environment was controlled, and the ambient temperature stayed relatively constant during the T-history test. Three-millimeter-diameter T-type thermocouple probes were used to measure the ambient temperature around the test tubes. The specifications and dimensions of the thermocouples and test tubes are shown in Table 2.

**Figure 2:**
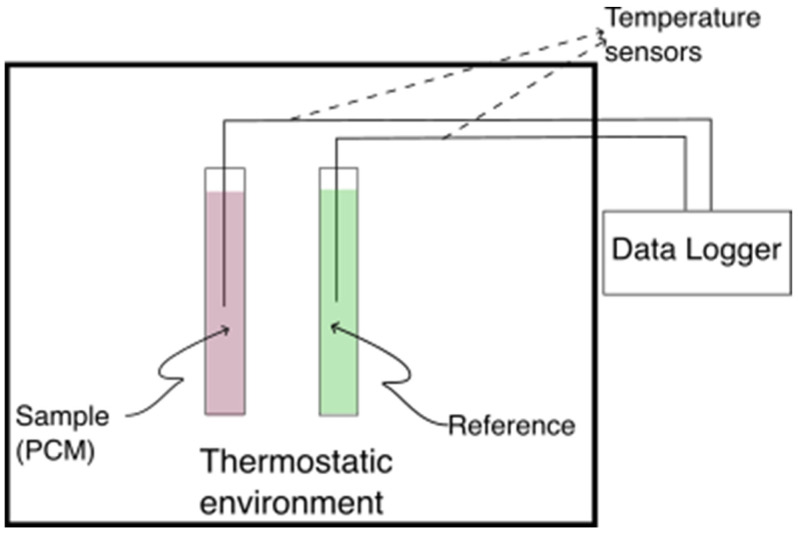
T-history test section schematic.

The motionless air environment was established in a 1000 mm × 400 mm × 300 mm polystyrene box with a 2 mm wall thickness, which was placed in an environmental chamber. The box was customized to accommodate thermocouples and test tubes according to the schematic in Figure 2. To ensure spatial temperature uniformity, five ambient temperature sensors were evenly distributed within the box. The uniformity of temperatures was assessed by calculating the standard deviation of the ambient temperatures measured by these sensors, which consistently remained within ±0.2 °C before the start of each solidification/melting process of the PCM.

### 2.2. Temperature Uncertainty Estimation

The accuracy of the temperature sensors significantly impacts the precision of PCM property estimations [25,33]. Therefore, all thermocouples were calibrated before the sample preparation, and an uncertainty analysis for temperature estimation was conducted. Calibration involved the use of a thermostatic bath filled with a 50% water–50% ethylene glycol solution, spanning temperatures from 0 °C to 40 °C in 10 °C intervals. A reference-probe thermometer (Dostmann electronic GmbH, Wertheim, Germany, P795) equipped with a PT100 probe (accuracy: ±0.015 °C) served as the standard. The thermostatic bath, set at 0 °C, acted as the external cold junction. During calibration, after stabilizing the bath temperature, more than 30 data points of millivolt readings from both the thermocouple under test and the external cold junction were recorded at each temperature level. A second-degree polynomial curve fit was applied to the voltage difference between the thermocouple under test and the external cold junction against the reference probe temperature. The individual standard uncertainties in measurements contributing to the total uncertainty in temperature estimation are presented in Table 3, following a similar analysis conducted by Upot et al. [33], which considers uncertainties from various sources of error during the thermocouple calibration process. The individual standard uncertainties $\left(\varepsilon_{individual}\right)$ mentioned in Table 3 are used to calculate the combined standard uncertainty $\left(\varepsilon_{combined}\right),\;$ as shown in Equation (1). The estimated combined standard uncertainty was ±0.23 °C.

(1) $$\varepsilon_{combined}=\sqrt{\sum \varepsilon_{individual}^{2}}$$

### 2.3. PCM Preparation

Two composites of SSD, 1E-3SPA and 2E-3SPA, were characterized in this study. 1E-3SPA and 2E-3SPA contain 12 wt.% and 9 wt.% NaCl, respectively, as listed in Table 4. Both composite compositions have the same content of nucleator (4 wt.% borax) and thickener (3 wt.% SPA). As per the guidelines in RAL standards [36], three samples of each composite were tested, each sample was cycled three times, and enthalpy–temperature curves for the third cycle were reported.

The composites under study were prepared by mixing the anhydrous sodium sulfate, anhydrous borax, SPA, NaCl, and distilled water in the weight ratios shown in Table 4. The weight of the material was measured with a weighing scale with an accuracy of ±0.1 mg.

**Figure 3:**
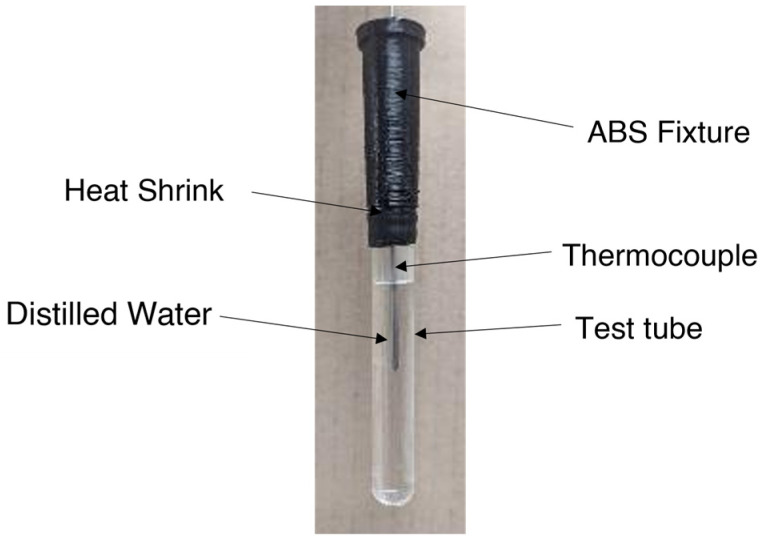
Reference material (distilled water) test tube.

Initially, the mixture was frozen at −4 °C in a freezer for 10 h and subsequently crushed into a fine powder using a mixer to ensure homogeneity. The ground mixture was then melted at 40 °C for 10 h in a forced convection oven, sealed in a polyethylene bag to prevent moisture loss. Before loading it into test tubes, the PCM was vigorously mixed to ensure uniformity. Samples were maintained at 40 °C during transfer to the test tubes. After loading the PCM, the test tubes were sealed using a heat-shrink material and secured with a fixture that held the thermocouples and the test tube together. The reference test tube with distilled water is illustrated in Figure 3.

### 2.4. Data Evaluation Methodology

As discussed in Section 1, the T-history method provides a more effective approach for characterizing salt hydrates than DSC. This method involves placing the PCM and reference material into separate test tubes, each equipped with a temperature sensor positioned at the tube’s center. Initially, both tubes are maintained at thermal equilibrium within a controlled environment. Subsequently, they are simultaneously exposed to a different ambient temperature, allowing each material to equilibrate with its surroundings over a predefined duration. Figure 2 illustrates a general schematic of the T-history experimental setup.

The test tube geometry and the ambient conditions ensure that a lumped capacitance assumption can be applied. By applying transient energy balance equations to the temperature–time data of the PCM and reference, the bulk thermophysical properties of PCM—such as $H_{f}$, $T_{m}$, and the degree of supercooling—are obtained. Before delving into the proposed data evaluation methodology, it is essential to review existing ones. Consequently, the discussion of this section is divided into two parts. The first part reviews the currently available data evaluation methodologies and discusses their advantages and limitations. The second part details the proposed data evaluation methodology.

#### 2.4.1. Current Data Evaluation Methodologies

**Figure 4:**
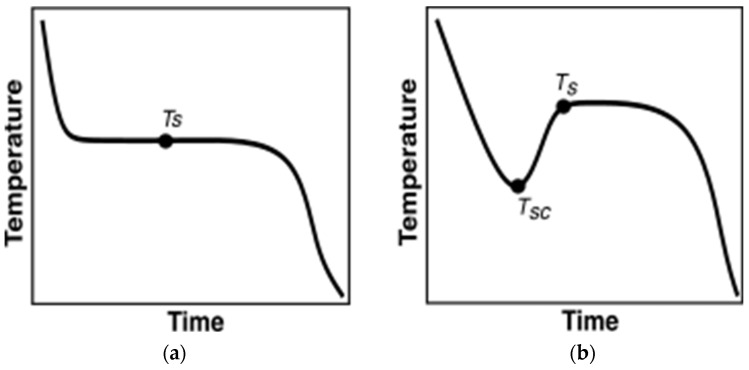
PCM behavior during cooling vs. time. (**a**) No supercooling; (**b**) with supercooling.

Before discussing the methodologies researchers implemented to characterize PCMs, it is important to discuss the variations in their temperature evolution trends. During the melting, almost all the PCMs heat up monotonously. However, not all PCMs monotonously cool down during solidification. Figure 4a depicts monotonously cooling PCM undergoing solidification. In contrast, as illustrated in Figure 4b, certain PCMs exhibit supercooling behavior, where temperature decrease is uninterrupted until reaching the supercooling temperature, T_SC_. Heat is then released until the PCM reaches the solidification temperature, $T_{S}$, which is higher than $T_{SC}$, after which phase change initiates, transitioning into a solid phase. Post T_s_, the PCM monotonously cools down.

Due to the distinctive supercooling behavior exhibited by some PCMs, careful consideration is necessary when analyzing T-history curves for these materials. The PCMs tested in the current paper are inorganic and demonstrate significant supercooling. Henceforth, further discussion will be focused on characterizing the PCMs that demonstrate supercooling.

Irrespective of the variations in the methodologies implemented by several studies, the equations governing the derivation of thermophysical properties of PCM using the T-history method are based on two primary assumptions, as listed by Tan et al. [37].

*First assumption:* The applicability of lumped thermal capacitance in the systems undergoing testing.

When a body at a uniform temperature T is suddenly exposed to an ambient temperature different from its initial temperature, one can assume a lumped capacitance model, meaning the thermal gradients within the system are negligible if the Biot number (Bi) is less than 0.1. Thus, it is critical to ensure that Bi is less than 0.1 in the experimental setup.

*Second assumption:* Equality of heat rate for PCM and reference at the same temperature difference.

The heat flow rate from the PCM, $q_{pcm}$ and reference, $q_{ref}$ are equal for the same temperature difference; i.e., when $T_{ref}-T_{\infty }=T_{pcm}-T_{\infty }\;$, then $q_{ref}=q_{pcm}$.

Since the test tubes are cylindrical, the resultant thermal resistance for the PCM ($R_{th,pcm})$ and reference ($R_{th,ref})$ assuming a simple 1D-radial heat transfer is given in Equations (2) and (3), respectively.

(2) $$R_{th,ref}=R_{th,conv,ref}+R_{th,cond,tube}=\frac{1}{h_{ref}A_{o}}+\frac{R_{o}\ln\left(\frac{R_{o}}{R_{i}}\right)}{k_{tube}A_{o}}$$

(3) $$R_{th,pcm}=R_{th,conv,pcm}+R_{th,cond,tube}=\frac{1}{h_{pcm}A_{o}}+\frac{R_{o}\ln\left(\frac{R_{o}}{R_{i}}\right)}{k_{tube}A_{o}}$$

where $R_{th,conv,ref}$ and $R_{th,conv,pcm}$ are the convective thermal resistances of the reference and PCM, respectively. $R_{th,cond,tube}$ is the conductive thermal resistance of the test tube. $R_{o}$ and $R_{i}$ are the outer and internal radii of the test tube, respectively. $A_{o}$ is the outer surface area of the test tube, and $k_{tube}$ is the thermal conductivity of the test tube material. Since the test tubes used to prepare both the reference and PCM have the same material and geometry, the conductive thermal resistance due to the tube is the same in both the PCM and reference.

According to the analysis performed by Badenhorst et al. [38] on an experimental setup similar to the one used in the current paper, the convective heat transfer coefficient value could be as high as 25 W·m^−2^·K^−1^. This suggests that the heat transfer mode can still involve natural convection around the tubes [39]. The thermal conductivity of the borosilicate is 1.14 W·m^−1^·K^−1^ [40]. Thus, the maximum value of the ratio of conductive and convective resistance $max(\frac{R_{th,cond,tube}}{R_{th,conv,ref}})$ for the geometry of the test tubes used in the current paper is 0.024 and is <<1. Thus, the conductive resistance due to the test tube can be neglected in Equations (2) and (3), respectively. Since convective thermal resistance is the dominant resistance in both the PCM and reference, the convective heat transfer coefficient and heat transfer area must be the same for both the PCM and reference for the same temperature difference to apply the second assumption.

Zhang et al. [25] proposed deriving the thermophysical properties of a PCM by analyzing its T-history profile in three zones: solid, liquid, and phase change. This method obtains the bulk thermophysical properties of the PCM, such as latent heat and the specific heat of the PCM in the liquid and solid phases. This method assumes that the convective heat transfer coefficient is constant and equal for both the PCM and reference systems in each zone. However, the convective heat transfer coefficient is a function of the difference between the surface temperature of the system and the ambient temperature [39]. In a T-history test, the reference and PCM temperature continuously change until they reach thermal equilibrium with the ambient. Thus, the constant heat transfer coefficient assumption does not have a sound physical basis.

Marín et al. [28] overcame the limitation of Zhang’s method by analyzing the T-history curves in very small intervals of temperatures and applying energy balance to these small intervals. This method assumes that convection is the dominant heat transfer mode and that, if the interval is small enough, the convective heat transfer coefficients are equal for both the PCM and reference if the average temperatures of the PCM and reference are the same in the interval.

**Figure 5:**
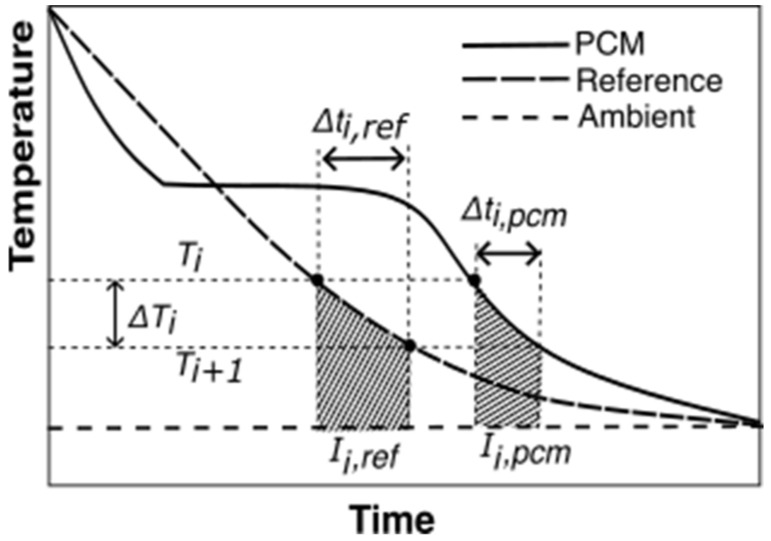
T-history data with an illustration of *ΔT_i_*, $\Delta t_{i,pcm},$ and ${\Delta t}_{i,ref}$ from Equations (6)–(8) for the Marin method. The shaded areas represent the area between the reference and ambient, $I_{i,ref}$, and, PCM and ambient temperature trends, $I_{i,pcm}$, for temperature interval, $\Delta T_{i}.$

The transient energy balance equations for the reference and PCM for the temperature interval $\Delta T_{i}\;$(from Figure 5) are shown in Equations (4) and (5), respectively.

(4) $${(m_{ref}C}_{p,ref}+{m_{tube}C}_{p,tube})\Delta T_{i}=-\left(hA_{o}\right)\int_{t_{i,ref}}^{t_{i,ref}+\Delta t_{i,ref}}\left(T_{ref,i}-T_{\infty ,i}\right)dt$$

(5) $${(m_{pcm}C}_{p,pcm}+{m_{tube}C}_{p,tube})\Delta T_{i}=-\left(hA_{o}\right)\int_{t_{i,pcm}}^{t_{i,pcm}+\Delta t_{i,pcm}}\left(T_{pcm,i}-T_{\infty ,i}\right)dt$$

where $m_{ref}$, $m_{tube},$ and $m_{pcm}$ are masses of the reference material, test tube, and PCM, respectively. $C_{p,\;ref}$, $C_{p,pcm},$ and $C_{p,tube}$ are specific heats of the reference material, PCM, and test tube material, respectively. $\Delta t_{i,pcm}$ and ${\Delta t}_{i,ref}$ are the time intervals for the PCM and the reference material, respectively, for a given temperature interval, $\Delta T_{i}=T_{i}-T_{i+1}$**.**

Applying equal convective heat transfer coefficient (h) for equal $\left(T_{ref,i}-T_{\infty ,i}\right)$ and $\left(T_{pcm,i}-T_{\infty ,i}\right)$, combined with the relationship between enthalpy change and specific heat for the PCM,$\;C_{p,pcm}\Delta T=\Delta H_{pcm}({\bar{T}}_{pcm,i})$, to Equations (4) and (5) provides Equation (6), which is the equation provided by Marín et al. [28].

(6) $$\begin{array}{c} \Delta H_{pcm}\left({\bar{T}}_{i,pcm}\right)=\left(\frac{m_{ref}C_{p,ref}\left({\bar{T}}_{pcm,i}\right)+m_{tube}C_{p,tube}\left({\bar{T}}_{pcm,i}\right)}{m_{pcm}}\right)\frac{I_{i,pcm}}{I_{i,ref}}\Delta T_{i}-\\ \frac{m_{tube}}{m_{pcm}}C_{p,tube}\left({\bar{T}}_{pcm,i}\right)\Delta T_{i}\; \end{array}$$

where ${\bar{T}}_{pcm,i}$ is the average PCM temperature of the interval under study. $I_{i,pcm}$ and $I_{ref,i}$ are the area under the PCM and reference temperature curves, respectively, calculated using Equations (7) and (8).

(7) $$\;I_{i,pcm}=\int_{t_{i,pcm}}^{t_{i,pcm}+\Delta t_{i,pcm}}\left(T_{pcm,i}-T_{\infty ,i}\right)dt$$

(8) $$\;I_{i,ref}=\int_{t_{i,ref}}^{t_{i,ref}+\Delta t_{i,ref}}\left(T_{ref,i}-T_{\infty ,i}\right)dt$$

An example temperature interval for a PCM cooling experiment is illustrated in Figure 5.

Once the enthalpy values are obtained for each interval, enthalpy–temperature curves can be obtained as a sum of the step enthalpy changes obtained for each temperature ${\bar{T}}_{pcm,i},$ as shown in Equation (9), where $H_{0}$ is the reference enthalpy, and N is the number of PCM intervals.

(9) $$H_{pcm}\left({\bar{T}}_{pcm,i}\right)=\sum_{i=1}^{N}\Delta H_{pcm}\left({\bar{T}}_{pcm,i}\right)+H_{0}$$

Marín et al. [28] reported enthalpy–temperature curves for n-Hexadecane using the methodology mentioned above. Many studies since then have reported enthalpy–temperature curves of different PCMs with no supercooling or insignificant supercooling using this methodology [41,42,43]. However, no explicit details have been provided on the data treatment of PCM with significant supercooling [35].

Sandnes and Rekstad [33] proposed a data evaluation method for materials that undergo significant supercooling. This method considers the heat flow rate of the reference, $q_{ref,i}$, in a temperature interval as the function of the difference between the reference and ambient temperatures, $\left({\bar{T}}_{ref,i}-{\bar{T}}_{\infty ,i}\right)$. They also considered the sensible heat change in the sensor in the energy balance. The computation of $q_{ref,i}$ in an interval $\Delta T_{int}$ including the sensor mass, $m_{sen}$, and the sensor’s specific heat, $C_{p,sen}$, is shown in Equation (10).

(10) $$q_{ref,i}={(m_{ref}C}_{p,ref}\left({\bar{T}}_{ref,i}\right)+{m_{tube}C}_{p,tube}\left({\bar{T}}_{ref,i}\right)+{m_{sen}C}_{p,sen}\left({\bar{T}}_{ref,i}\right))\frac{\Delta T_{i}}{\Delta t_{i,ref}}$$

A second-degree polynomial was implemented to calculate $q_{ref,i}$ for each time interval $\Delta t_{i,ref}$ as a function of $\left({\bar{T}}_{ref,i}-{\bar{T}}_{\infty ,i}\right)$. In alignment with the second assumption of an equal heat flow rate for the same temperature difference, the polynomial was used to calculate the heat flow rate in the PCM in each interval ${q\;}_{pcm,i}$ as a function of $\left({\bar{T}}_{pcm,i}-{\bar{T}}_{\infty ,i}\right)$. Once $q_{pcm,i}$ is determined, the step enthalpy change is determined using Equation (11).

(11) $$\Delta H_{pcm}\left({\bar{T}}_{pcm,i}\right)=\frac{{q_{pcm,i}\Delta t_{i,pcm}-(m}_{sen}C_{p,sen}\left({\bar{T}}_{pcm,i}\right)+m_{tube}C_{p,tube}\left({\bar{T}}_{pcm,i}\right))(\Delta T_{i})}{m_{pcm}}$$

Sandnes and Rekstad [33] used their methodology to generate solidification enthalpy–temperature curves of disodium hydrogen phosphate dodecahydrate, SAT, and modified STL-47, a commercially available PCM. However, this methodology lacks a sound thermodynamic basis for using the polynomial for curve fitting the heat transfer rate.

D’Avignon et al.’s work [35] is one of the few studies that elaborated on T-history data treatment for PCMs with significant supercooling. They recommended using absolute temperature intervals to display supercooling in the enthalpy versus temperature curve of the PCM. Since additional temperature intervals are required to capture supercooling behavior, the number of PCM temperature intervals will be higher than the reference temperature intervals. D’Avignon et al. [35] pointed out the increased PCM temperature intervals. However, no further information was provided on addressing the issues.

Tan et al. [37] proposed an algorithm using MATLAB v2020b that smoothed the temperature–time data of the PCM and reference material using splines from the Shape Language Modeling (SLM)-based toolbox. They considered both positive and negative changes in temperature for calculating the temperature intervals. The reference temperature–time curve was curve fit using splines to interpolate reference time values for each PCM interval, as shown in Equation (12).

(12) $$\Delta t_{i,ref}=t_{i+1,ref}-t_{i,ref}=f\left(T_{pcm,i+1}\right)-f\left(T_{pcm,i}\right)$$

To avoid over-fitting, smoothing was used only for a specific temperature range that captured the PCM’s latent behavior during melting. No smoothing was implemented for the PCM solidification data. However, using splines lacks sound thermodynamics-based reasoning, and selecting a temperature range to smoothen incorporates subjectivity.

In summary, Equation (6) provided by Marín et al. [28] forms the foundation for generating enthalpy–temperature curves for PCM. Although their study is well suited for PCMs without supercooling, they did not provide an explicit data evaluation procedure for PCMs with supercooling. Sandnes and Rekstad [33] proposed a data evaluation algorithm suitable for PCMs with supercooling. However, this method lacks sound thermodynamics-backed reasoning for using polynomials for curve fitting the heat transfer rate. D’Avignon et al. [35] proposed a method of scoping for temperature intervals using the absolute temperature difference, suitable for PCMs with supercooling, which leads to an increased number of PCM temperature intervals than the reference’s. However, they did not elaborate on the issues raised by using their method. Tan et al. [37] proposed a data evaluation algorithm that uses splines to interpolate the entire reference temperature–time data and PCM melting data during phase change. However, using splines lacks a sound thermodynamic basis, and selecting a particular temperature range for PCM data interpolation incorporates user bias into the derived data. Therefore, the authors in this paper proposed a methodology that considers both positive and negative changes in temperature, suitable for PCMs demonstrating significant supercooling along with a curve fit method based on the physics driving the T-history process without any user-induced bias.

#### 2.4.2. Proposed Data Evaluation Methodology

**Figure 6:**
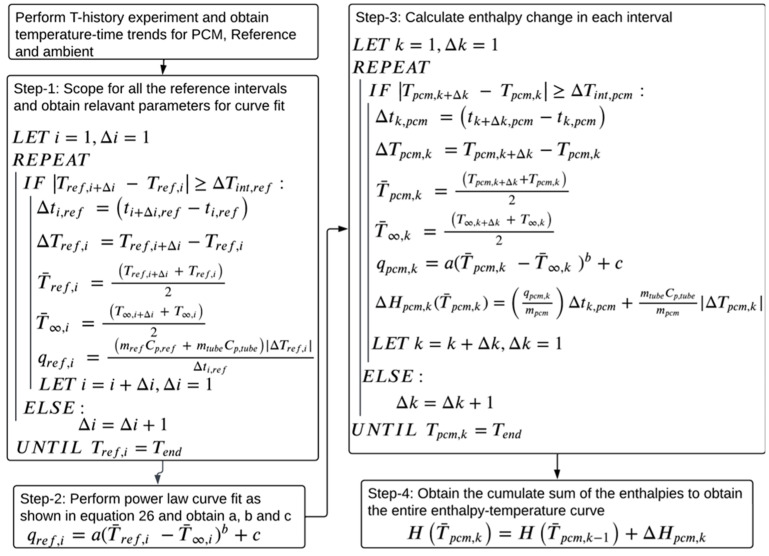
Algorithm of the proposed methodology.

For the current study, the authors propose a variation on the method proposed by Sandnes and Rekstad [33], in which a power law-based curve fit is used to estimate the reference material heat transfer rate as a function of the temperature difference between the reference and the ambient instead of a polynomial. The details of the proposed methodology and the physics-driven reasoning behind using a power law are discussed step-by-step. The algorithm for the proposed data evaluation method is illustrated in Figure 6.

To select a suitable temperature interval, Tan et al. [37] and Stanković et al. [44] implemented a flexible temperature interval condition $|T_{i+\Delta i}-T_{i}|\geq \Delta T_{int}$ during data treatment, where $\Delta T_{int}$ is the minimum allowed temperature interval. A similar condition is used in the current paper to scope for reference and PCM temperature intervals. As suggested by D’Avignon et al. [35], the absolute value of the temperature difference, $\left|T_{i+\Delta i}-T_{i}\right|$, is considered to capture the supercooling behavior of the PCM.

Step 1: Compute $\Delta T_{i,ref}$ and $\Delta t_{i,ref}$ for each reference temperature interval, using a minimum allowed reference temperature interval $\Delta T_{int,ref}$ in the following condition, $|T_{ref,i+\Delta i}-T_{ref,i}|\geq \Delta T_{int,ref}$. To make the condition equally applicable to cooling and heating cases, the absolute value of $T_{ref,i+\Delta i}-T_{ref,i}$ is used.

Compute the average reference and ambient temperatures, ${\bar{T}}_{ref,i}\;$ and ${\bar{T}}_{\infty ,i}$, for each reference temperature interval using the trapezoid rule, as shown in Equations (13) and (14), respectively.

(13) $$\;\;{\bar{T}}_{ref,i}=\frac{1}{\Delta t_{i,ref}}\int_{t_{i,ref}}^{t_{i,ref}+\Delta t_{i,ref}}T_{ref}dt=\left(T_{ref,t_{i,ref}+\Delta t_{i,ref}}+T_{ref,t_{i,ref}}\right)/2$$

(14) $$\;{\bar{\;T}}_{\infty ,i}=\frac{1}{\Delta t_{i,ref}}\int_{t_{i,ref}}^{t_{i,ref}+\Delta t_{i,ref}}T_{\infty }dt=\left(T_{\infty ,t_{i,ref}+\Delta t_{i,ref}}+T_{\infty ,t_{i,ref}}\right)/2$$

As shown in Equation (15), compute the reference heat transfer rate for each interval, $q_{ref,i}$. Implementing an absolute value of $\Delta T_{i,ref}$ in Equation (15) keeps $q_{ref,i}$ positive across heating and cooling cases.

(15) $$\;{q_{ref,i}=(m_{ref}C}_{p,ref}+{m_{tube}C}_{p,tube})\frac{\left|\Delta T_{i,ref}\right|}{\Delta t_{i,ref}}$$

Step 2: Since the ambient condition is motionless air, heat transfer is driven by natural convection. Natural convection is a buoyancy-driven heat transfer process, and previous studies show that the convective heat transfer coefficient h is proportional to the temperature difference between the surface and the ambient and could be correlated through power law ${\left(T_{ref}-T_{\infty }\right)}^{n}$ [39]. Thus, the heat transfer rate in a temperature interval is correlated to $\left({\bar{T}}_{ref,i}-{\bar{T}}_{\infty ,i}\right)$ through a power law, making the correlation physics-driven. $q_{ref,i}$ and $\left({\bar{T}}_{ref,i}-{\bar{T}}_{\infty ,i}\right)$ from all the reference intervals obtained in step 1 are fit to the power law, as shown in Equation (16), to obtain its coefficients *a*, *b*, and *c*. The assumption is that *a*, *b*, and *c* are independent of temperatures and remain constant over all the temperature intervals, and that they are identical for both the PCM and reference systems.

(16) $$q_{ref,i}={a\left({\bar{T}}_{ref,i}-{\bar{T}}_{\infty ,i}\right)}^{b}+c$$

Step 3: Obtain PCM temperature intervals using $|T_{pcm,k+\Delta k}-T_{pcm,k}|\geq \Delta T_{int,pcm}$. Similar to step 1, obtain $\Delta T_{pcm,k},\;\Delta t_{pcm,k}$ and the average PCM and ambient temperatures, ${\bar{T}}_{pcm,k}\;$ and ${\bar{T}}_{\infty ,k}$, respectively, for each interval using the trapezoid rule. Then, for each interval, compute the PCM heat transfer rate, $q_{pcm,k}$, using $\left({\bar{T}}_{pcm,k}-{\bar{T}}_{\infty ,k}\right)$ and the power law curve fit parameters obtained in step 2, as shown in Equation (17). Compute the enthalpy change for each PCM interval ${\Delta H}_{pcm}\left({\bar{T}}_{pcm,k}\right)$ using Equation (18). The absolute value of $\left|\Delta T_{pcm,k}\right|\;$ is used to follow an assumption that the heat released in the PCM during the supercooling process and heat transferred to the ambient are the same.

(17) $$q_{pcm,k}={a\left({\bar{T}}_{pcm,k}-{\bar{T}}_{\infty ,k}\right)}^{b}+c$$

(18) $$\;{\Delta H}_{pcm}\left({\bar{T}}_{pcm,k}\right)=\left(\frac{q_{pcm,k}}{m_{pcm}}\right)\Delta t_{k,pcm}-\frac{m_{tube}}{m_{pcm}}C_{p,tube}|\Delta T_{pcm,k}|$$

Step 4: Once ${\Delta H}_{pcm}\left({\bar{T}}_{pcm,k}\right)$ is obtained for each PCM interval, their cumulative sum is calculated to obtain an enthalpy–temperature curve.

## 3. Results and Discussion

This section is divided into five subsections. Since validation with standard test materials is an important step to verify the suitability of the test setup and data analysis method, in the first subsection, we present the enthalpy–temperature curves of n-Hexadecane generated using the proposed method. The methodology is validated by comparing the bulk temperature properties obtained from enthalpy–temperature curves of n-Hexadecane with the DSC and T-history results published in the literature. In the second subsection, we present the enthalpy–temperature curves and the bulk thermophysical properties of the two SSD composites, 1E-3SPA and 2E-3SPA. In the third subsection, the thermophysical properties of the composites are summarized and the volumetric energy storage densities of the two composites are compared to the commercially available PCMs with similar melting ranges of the composites. In fourth subsection, the convective heat transfer coefficients are calculated to verify the applicability of the lumped capacitance assumption to the current setup. In the fifth subsection, the uncertainty analysis of the measurement technique is presented.

### 3.1. Validation of Proposed Methodology with n-Hexadecane’s Data Reported in the Literature

n-Hexadecane is a pure paraffin material used as a PCM in TES [45,46,47] and widely used as a standard material. n-Hexadecane’s solidification properties obtained from DSC are compared to the thermophysical properties obtained from the T-history to validate the T-history test setup in past studies [28,41,48]. Similarly, in the current study, the solidification properties of n-Hexadecane were computed and compared to the values reported in the literature as a validation step.

**Figure 7:**
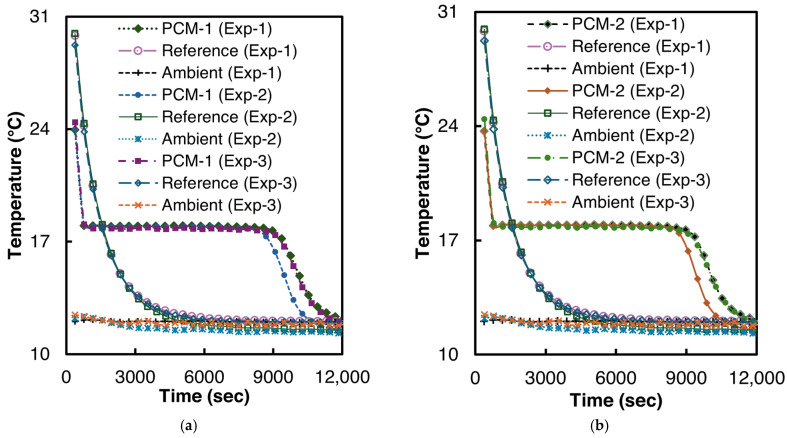
Temperature vs. time curves for n-Hexadecane solidification (**a**) PCM-1, (**b**) PCM-2.

The solidification experiments were performed at ambient temperatures of 11–12 °C. Two samples of n-Hexadecane were prepared for the experiment, each with 6.3 g of n-Hexadecane. To ensure repeatability, both samples were tested thrice. Before the start of every test, both reference and PCM test tubes were placed in a thermostatic bath at 40 °C for at least six hours to reach thermal equilibrium. The T-history curves of the solidification experiments are illustrated in Figure 7.

As can be seen from Figure 7, n-Hexadecane demonstrated a very low supercooling of 0.2 °C in all the experiments. The solidification temperature for n-Hexadecane is 17.96 °C (σ = 0.02 °C), which is in good agreement with the 17.75–18.1 °C reported by Paola et al. [48].

**Figure 8:**
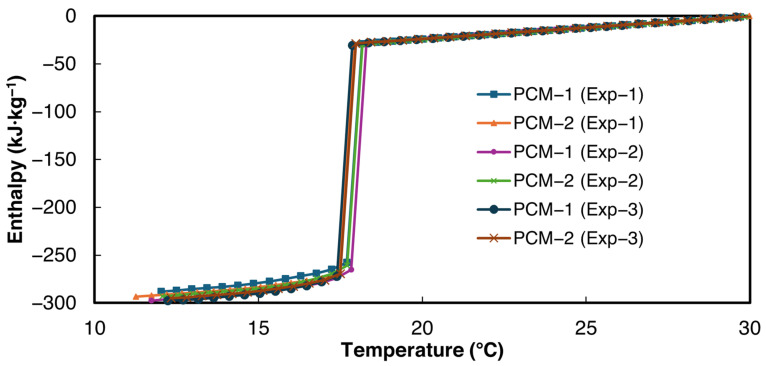
Solidification enthalpy–temperature curve of n-Hexadecane, according to the current study.

The enthalpy–temperature curves obtained using the power law curve fit method proposed in this paper are shown in Figure 8. For the validation studies, the minimum allowed temperature interval $\Delta T_{int,pcm}=\Delta T_{int,ref}=$ 0.2 °C was used. For the enthalpy temperature curve, 30 °C was chosen as a reference for zero enthalpy. The enthalpy–temperature curves demonstrate close agreement with each other.

The large enthalpy drop at the solidification temperature is computed as the latent heat of solidification. The slope of the enthalpy–temperature data above 20 °C was taken as liquid-state specific heat, and the slope of the enthalpy–temperature data below 14 °C was taken as the solid-state specific heat of the PCM. The average values of the bulk thermophysical properties and their respective standard deviations obtained from the current study are presented in Table 5. As can be seen from Table 5, the latent heat values and liquid-state specific heat reported in the current study are in very close agreement with the DSC values reported by Velez et al. [49], thereby validating the experimental setup and data evaluation method. Velez et al. [49] reported the solid-state specific heat only for temperatures below 8 °C. Therefore, the solid-state specific heat was not used for calculating the deviation.

In the current study, the solidification experiments were performed at an ambient temperature of 12 °C. However, Marín et al. [28] and Paola et al. [48] maintained 0 °C and 10 °C ambient temperatures, respectively, and reported a latent heat of solidification of 252 and 239.8 kJ·kg^−1^, respectively. Since the difference in n-Hexadecane’s phase transition temperature and the ambient temperature is the lowest in the current study, the cooling rates are slower in comparison to the two studies mentioned above. Also, the lower temperature difference in the current study reduces the Bi, which could be attributed to the lower deviation from the DSC values.

### 3.2. Salt Hydrate Composites

As per RAL standards [36], three samples of each of the two salt hydrate composites, 1E-3SPA and 2E-3SPA (see Table 4), were cycled three times, and the last cycle of T-history data was used to generate the enthalpy–temperature curves. The samples were tested at 5 and 43 °C ambient temperatures in cooling and heating, respectively, with consecutive solidification and melting, and the resultant enthalpy–temperature curves are presented in the following sections.

#### 3.2.1. 1E-3SPA

**Figure 9:**
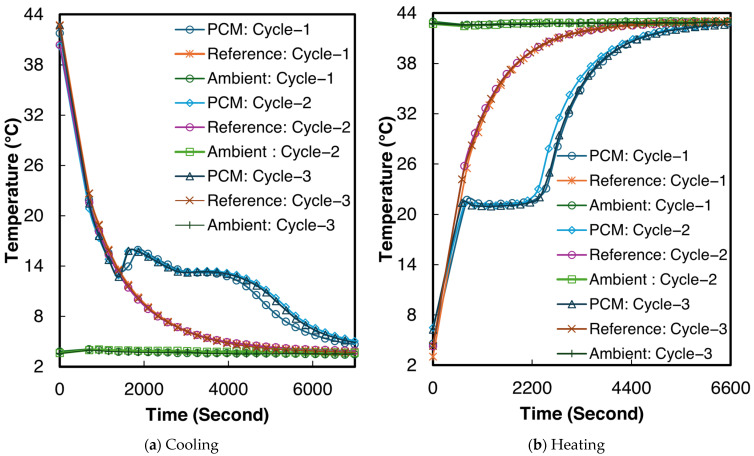
Temperature vs. time curves for sample 2 of 1E-3SPA.

The temperature–time curves for sample 2 of the 1E-3SPA composite during all three melting and cooling processes are shown in Figure 9. From Figure 9a,b, supercooling and solidification temperatures can be determined, but the end of solidification and melting are not clear. The inflection point of the temperature derivative of time can be adopted as the end of the phase change [50]. The inflection points were identified numerically to determine the end of solidification and melting, respectively. When averaging the values across all three tested samples, the 1E-3SPA composite exhibits a supercooling degree of 1.6 °C, with the start of solidification at 12.8 °C and the end of solidification at 9.4 °C. The onset of melting occurs at 21.3 °C and ends at 26.2 °C. In the current work, temperature hysteresis is defined as the temperature difference between the melting onset and the solidification temperature. The 1E-3SPA composite shows a hysteresis of 7.1 °C. Table 6 summarizes all the temperature characteristics of 1E-3SPA for cycle 3 of the studied samples.

**Figure 10:**
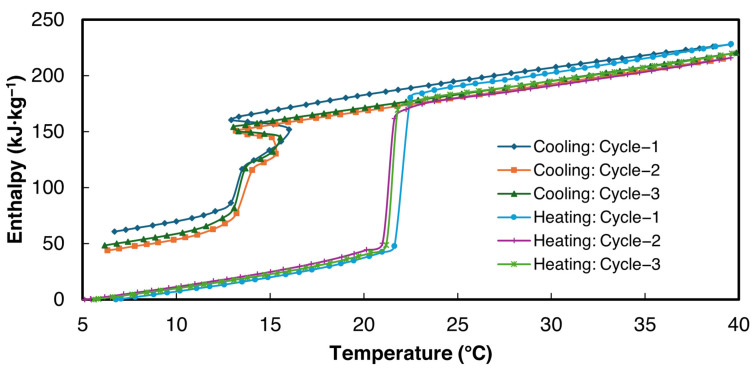
Enthalpy vs. temperature curves for sample 2 of 1E-3SPA.

The enthalpy curves for all three cycles were derived from the proposed power law method within the temperature range of 5–40 °C. These are presented in Figure 10 for sample 2 of 1E-3SPA. $\Delta T_{int}$= 0.2 °C was used for the data analysis.

The bulk thermophysical properties obtained from enthalpy–temperature curves for all three samples for cycle 3 in Figure 10 are summarized in Table 7. As can be seen in Figure 10, the slopes of the enthalpy data in solid and liquid phases are similar, showing that the solid-state specific heat values obtained from heating and cooling cases are the same. Since more data points are available in the heating enthalpy–temperature curve for both solid and liquid phases, heating data were used to determine the specific heat. Specific heats in liquid and solid states were obtained by applying a linear curve fit to the enthalpy data above and below the melting zone. The enthalpy hysteresis values, which signify thermal and structural irreversibilities, shown in Table 7, are defined as the enthalpy difference between the cooling and heating enthalpy curves at the end of the solidification temperature.

When filled to the 8-milliliter mark, all three samples weighed 12.9 g. Therefore, the approximate density of the composite is 1.65 g·cc^−1^. 1E-3SPA has a latent heat of fusion of 139 kJ·kg^−1^, resulting in a volumetric energy density of 1E-3SPA at 229.5 MJ·m^−3^, which is 27.5% higher than n-Hexadecane’s 180 MJ·m^−3^. The $T_{m}$ of 1E-3SPA is lowered due to the addition of 12% NaCl, bringing it to 21.3 °C, making it suitable for HVAC applications. However, the temperature hysteresis (7.1 °C) is quite large, which is not favorable for the thermal performance of TES.

#### 3.2.2. 2E-3SPA

**Figure 11:**
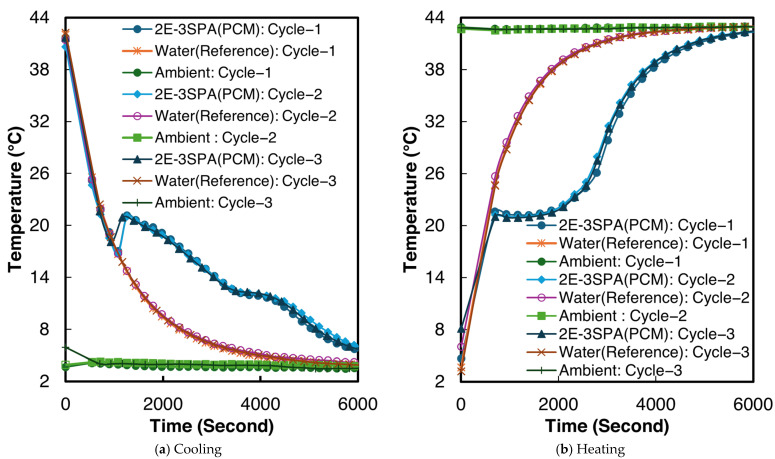
Temperature vs. time curves for sample 2 of 2E-3SPA.

The temperature–time curves for sample 2 of 2E-3SPA for all three melting and cooling processes are shown in Figure 11. The temperature characteristics for 2E-3SPA are summarized in Table 8. Although 2E-3SPA contains 3% lower NaCl than 1E-3SPA, the 2E-3SPA composite has a melting onset of 21.4 °C, similar to 1E-3SPA’s. The melting end point of 2E-3SPA is 27.9 °C. The supercooling temperature is 17.6 °C with a 1.9 °C degree of supercooling and a temperature hysteresis of 2 °C. The solidification end was 9.6 °C.

**Figure 12:**
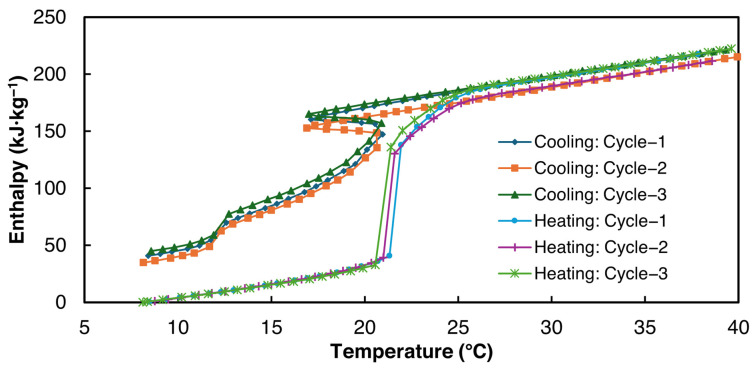
Enthalpy vs. temperature curves for 2E-3SPA.

The enthalpy curves for all three cycles were derived from the proposed power law fitting method using $\Delta T_{int}$= 0.2 °C for the data analysis within the temperature range of 8–40 °C and are presented in Figure 12. Table 9 summarizes the average properties of all three 2E-3SPA samples derived from the enthalpy–temperature curves. Similar to 1E-3SPA, the specific heat values in liquid and solid phases for 2E-3SPA were obtained using the heating T-history data.

As can be seen from Table 9, the average latent heat value for 2E-3SPA is 171 kJ·kg^−1^ during melting, 23% higher than 1E-3SPA’s. The densities of 1E-3SPA and 2E-3SPA were found to be equal. Thus, 2E-3SPA has a lucrative volumetric energy storage density of 282 MJ·m^−3^, 55% higher than that of n-Hexadecane, with readily available additives to tune the properties of SSD. While the large solidification range of 8 °C could be unfavorable for HVAC applications, the low hysteresis of 2E-3SPA is favorable.

### 3.3. Calculating Convective Heat Transfer Coefficient

For the lumped capacitance assumption to hold, the Biot number (Bi) must be less than 0.1. The convective heat transfer coefficient h is a key factor in calculating Bi. However, most studies in the literature fail to report the value of Bi. Cabeza and Badenhorst [38] analyzed various T-history methods and concluded that the h value ranges between 10 and 25 W·m^−2^·K^−1^. Thus, it is critical to compute the degree of variation in the h in our experiments to ensure the validity of the lumped capacitance assumption. To calculate the convective heat transfer coefficient from the obtained T-history data, the differential form of the transient energy balance equation, as shown in Equation (19), is used.

(19) $$h=-\frac{m_{ref}C_{p,ref}+m_{tube}C_{p,tube}}{A_{o}\left(T_{ref}-T_{\infty }\right)}\frac{dT_{ref}}{dt_{ref}}$$

**Figure 13:**
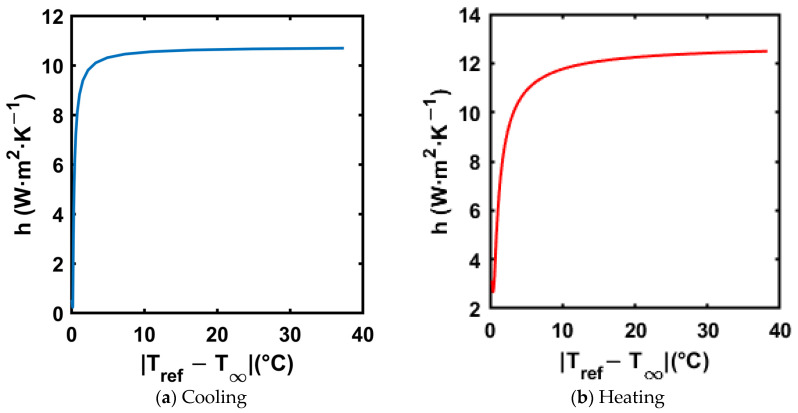
Convective heat transfer coefficient vs. ${|T}_{ref}-T_{\infty }|$ for reference material for both cooling and heating processes.

An exponential curve fit was applied to the reference material (distilled water) temperature–time trend to calculate the instantaneous temperature gradients in Equation (19). The resultant trends of the convective heat transfer coefficient for both cooling and heating processes are shown in Figure 13. Figure 13a,b show the trend of convective heat transfer coefficient in both cooling and heating, respectively.

The trends in Figure 13 show that the convective heat transfer coefficient is highest at the start of the test, as the heat transfer is driven by natural convection, and the temperature difference is at its highest at the start of the test. Also, the heat transfer coefficient value stays below 11 W·m^−2^·K^−1^ in cooling and 13 W·m^−2^·K^−1^ in heating, respectively. Bi  is calculated using Equation (20).

(20) $$\;\;\;\;Bi=\frac{hR_{o}}{2k_{ref}}$$

where h is the convective heat transfer coefficient, $R_{o}$ is the outer radius of the test tube, and $k_{ref}$ is the thermal conductivity of the reference material (distilled water). For distilled water, the thermal conductivity is 0.598 W·m^−1^·K^−1^, and the outer radius of the test tube is 6.35 mm. Thus, when using the highest convective heat transfer coefficient in the respective tests, with the maximum value of Bi for the heating process, the values are 0.058 and 0.053 for cooling, which are much below 0.1, satisfying the lumped capacitance assumption. Salt hydrates typically have a thermal conductivity range of 0.5–1.5 W·m^−1^·K^−1^ [51], holding the lumped capacitance assumption for the composites tested in the current paper.

### 3.4. Uncertainty Analysis

The T-history data is non-linear and transient in nature, and the data evaluation methodology consists of a curve-fitting procedure. Therefore, using the standard uncertainty propagation calculation to estimate the uncertainty of enthalpy and derived bulk thermophysical properties is not straightforward. Per the Joint Committee for Guides in Metrology (JCGM) recommendation [52], Monte Carlo simulations were carried out to estimate the uncertainty in the enthalpy change and specific heats. The Monte Carlo method estimates the output quantity’s probability density function (PDF) by propagating the input quantity’s PDF through the data evaluation algorithm. The output quantity PDF could be used to obtain the best estimate of the output quantity and its standard uncertainty. A total of one million simulations were performed for each item of T-history data to obtain significant digits below 1 kJ·kg^−1^ for enthalpy increment uncertainty estimation. As can be seen from Equation (6), the step change in enthalpy requires the following inputs, $m_{pcm}$, $m_{ref}$, $m_{tube}$, $C_{p,ref}$, $C_{p,tube}$, $T_{pcm}$, $T_{ref},$ and $T_{\infty }$. The probability density functions applied to the input quantities are summarized in Table 10. Conservative estimates of ±0.1 g and ± 0.1 kJ·kg^−1^·K^−1^ were applied for mass measurement and estimated specific heats of the reference material (distilled water) and test tube material (borosilicate glass), respectively. $\Delta T_{int}=0.2\;{}^{\circ}C$ was chosen for the Monte Carlo simulations for the estimation of uncertainty. Only the enthalpy data for cycle 3 for all three samples were considered.

**Figure 14:**
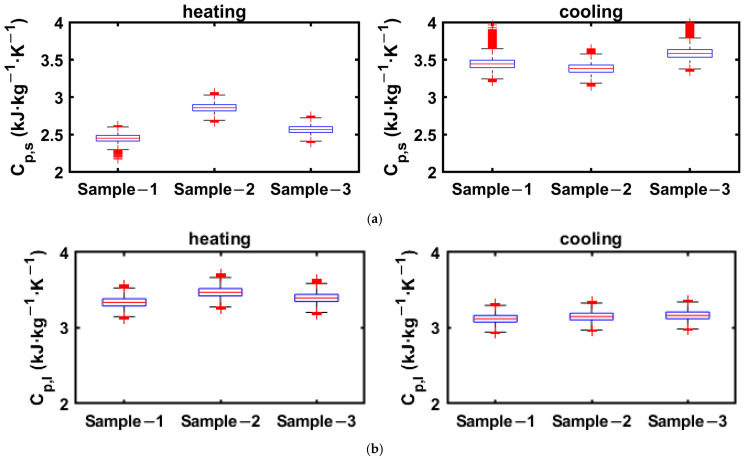
Solid- and liquid-state specific heats for 2E3SPA obtained from cooling and heating enthalpy curves. (**a**) Solid-phase specific heat: 2E-3SPA; (**b**) liquid-phase specific heat: 2E-3SPA.

The ranges of specific heats in solid and liquid phases obtained from the cooling and heating enthalpy curves of 2E-3SPA are presented in Figure 14, where the data greater than 1.5 times the IQR are taken as the outliers.

As can be seen Figure 14a, the number of outliers for the solid-phase specific heat from cooling data is much higher because the end of solidification is around 9 °C, and the enthalpy data below the solidification temperature is used to linearly curve fit between enthalpy and temperature to calculate the specific heat. Since there are fewer data points between 9 °C and the lowest temperature of the PCM in the experiment, the slope obtained from linear regression has a more pronounced effect on the added uncertainties. However, the number of outliers is lower for the estimation of solid-state specific heat from the heating process, as the onset of melting is around 21 °C, and enthalpy data between 5 and 21 °C are used to obtain the solid-state specific heat. Thus, it is suggested that the solid-state specific heat be obtained from the heating process enthalpy data. Since more data points are available for estimating the liquid-phase specific heat in both cooling and heating processes, it is less susceptible to added uncertainties. The whiskers for solid- and liquid-state specific heats obtained from heating data extend to ±0.3 kJ·kg^−1^.

**Figure 15:**
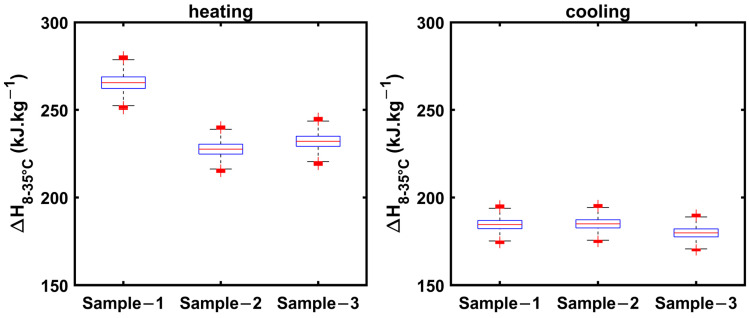
Enthalpy increment from 8 to 35 °C for 2E-3SPA obtained from cooling and heating data.

The uncertainty in estimating enthalpy increases from 8 to 35 °C, which encompasses specific heats in solid and liquid phases, as well as latent heat regions, for 1E-3SPA and 2E-3SPA in both cooling and heating processes. These are presented in Figure 15. The whiskers extend to ±15 kJ·kg^−1^ for the heating process and ±13 kJ·kg^−1^ for the cooling process.

The estimated values of the thermophysical properties and their associated uncertainties are summarized in Table 11. From Table 11 and Table 12, it can be concluded that the sample-to-sample variation is higher than the uncertainty in the estimation, which is a good indication of the measurement process. Larger variation from sample to sample can be attributed to the inhomogeneity of the composite.

### 3.5. Comparison of the Volumetric Energy Densities with State of the Art

The relevant thermophysical properties of 1E-3SPA and 2E-3SPA required for TES design and decision-making in the selection of PCM, obtained from their respective enthalpy–temperature curves, are presented in Table 12. The melting range of the 2E-3SPA falls well into the optimum melting range of the PCMs, 18–25 °C [9,10,11], required for HVAC applications in buildings, including data centers. Even after the addition of multiple additives, the volumetric energy storage density of 2E-3SPA is 55% higher than n-Hexadecane, a widely used organic PCM for HVAC applications. The degree of supercooling is also low at 2 °C, making this a lucrative PCM for HVAC applications in buildings.

The volumetric energy densities of selected PCMs were compared with state-of-the-art commercial PCMs that have melting points between 21 and 27 °C, similar to 2E-3SPA, as shown in Table 13. Table 13 shows that 2E-3SPA has a reasonably higher volumetric energy density compared to the commercial PCMs with a similar melting point range. However, SP25E2, a salt hydrate PCM by Rubitherm [53], has 2.1% higher volumetric energy densities than 2E-3SPA.

## 4. Conclusions and Future Work

PCMs are energy storage materials used in TES devices for energy savings and peak load shifting in HVAC applications. To commercialize the TES technology, the cost of TES devices needs to be reduced, which calls for the development of reliable and low-cost PCMs. SSD is a low-cost salt hydrate PCM. However, it melts at 32 °C, and for its use in HVAC applications, the melting point needs to be lowered by at least 10 °C.

For the current study, adhering to RAL standards, three samples of each of the two composites were tested through their T-history, and enthalpy–temperature curves were developed. The composites were 1E-3SPA and 2E-3SPA, each with 12 wt.% and 9 wt.% NaCl, respectively, for melting point suppression, and with 3 wt.% SPA as a thickener and 4 wt.% borax for supercooling reduction. The following conclusions can be drawn from the current study:

1. A modified data evaluation methodology was introduced to analyze the T-history data and obtain the enthalpy–temperature curves of the above-mentioned composite PCMs using Marin’s method. The modified methodology is built upon the physics-driven principles of heat transfer, suitable for PCMs demonstrating significant supercooling.
2. Using the proposed methodology, we found the following:
  - 1E-3SPA was found to have 139.1 kJ·kg^−1^ of latent heat with a melting onset temperature of 21 °C and a supercooling degree of 1.5 °C. However, it exhibited a large hysteresis of 7.7 °C, making it a less favorable PCM despite its lower cost.
  - 2E-3SPA was found to have 170.9 kJ·kg^−1^ of latent heat with a melting temperature of 21 °C and a supercooling degree of 2 °C. It showed a low hysteresis of 2 °C, making it a favorable PCM for HVAC applications. Moreover, the melting range of 2E-3SPA falls in the optimum melting temperature of PCMs for HVAC applications such as data centers.
  - The volumetric energy storage density of 2E-3SPA is 282 MJ·m^−3^, which is very close to the United States’ DOE target of 288 MJ·m^−3^ and is also closer to the volumetric energy storage densities of several commercial PCMs with similar melting ranges.
3. It can be concluded that the temperature characteristics and energy and bulk thermophysical properties are sensitive to the amount of NaCl in the composite.
4. Uncertainty estimation of the enthalpy and specific heat of both 1E-3SPA and 2E-3SPA was carried out using Monte Carlo simulations. The maximum deviation in the estimation of specific heat was ±0.3 kJ·kg^−1^·K^−1^, and in the estimation of the enthalpy increase from 8 to 35 °C, it was ±15 kJ·kg^−1^. It was found that the uncertainty in temperature estimation has the greatest impact on the uncertainty in estimating the PCM thermophysical properties. Thus, reduced uncertainty in temperature can reduce the uncertainties of the estimations further.

Future work on the PCMs studied in this research should focus on minimizing sample-to-sample variation in the estimation of latent heat of the composites by tightening and standardizing the preparation approach. Additional efforts should include analyzing the thermal conductivity of the PCMs using commercially available devices, evaluating the stability and long-term serviceability of the composites, and assessing material compatibility with containment structures in TES applications to identify suitable materials. Furthermore, the composite PCM should be integrated into a TES system to compare its performance with state-of-the-art TES technologies. The authors have already taken steps to investigate some of these aspects and intend to share their findings with the research community in the future.

## Figures and Tables

**Table 1:** SSD composites explored in the literature.

**Ref.**	[14]	[19]	[20]	[21]	[22]	[23]	[24]
**Method**	DSC						
**Base PCM**	Pure SSD	50 wt.% SSD	20 wt.% SSD	82 wt.% SSD	74.5 wt.% SSD	SSD	93 wt.% SSD
**Melting point suppressing agent**	-	50 wt.% Na_2_CO_3_·10H_2_O	80 wt.% Na_2_HPO_4_·12H_2_O	5 wt.% KCl	10 wt.% KCl and 10 wt.% NaCl	5% wt.% NaCl	-
**Nucleating agent**	-	-	2.5 wt.% Na_2_SiO_3_.9H_2_O	5 wt.% borax	3 wt.% borax	-	4 wt.% borax
**Thickener**	-	-	30 wt.% SiO_2_	5 wt.% PAM	1.5 wt.% CMC	CMC	3 wt.% SPA
**Thermal** **conductivity enhancer**	-	-	-	3 wt.% expanded graphite	1 wt.% carbon	-	-
$T_{m}$ **(°C)**	32	25	25	23.6	13.4	25	32
$H_{f}$ **(kJ·kg^−1^)**	238	187	143	111	128	-	227
**PCM type**	Pure	Eutectic	Composite				

**Table 2:** Thermocouple and PCM holder parameters.

Component	Parameter	Value
Thermocouple	Type	K
	Probe length (mm)	150
	Probe diameter (mm)	1.5
	Tip diameter (mm)	0.2
Test tube	Material	Borosilicate
	Outer diameter (mm)	12.7
	Wall thickness (mm)	1
	Length (mm)	100

**Table 3:** Uncertainty parameters for temperature estimation.

Uncertainty Component	Standard Uncertainty (°C)
Reference probe accuracy	0.015
Reference readout accuracy	0.002
Reference probe drift	0.0025
Thermocouple standard deviation	0.067
External cold junction standard deviation	0.003
Standard deviation of the calibration bath	0.002
DAQ measurement accuracy	0.02
Radial temperature uncertainty	0.02
Maximum deviation in temperature from the curve fit	0.22

**Table 4:** Compositions of the SSD composites.

Composite PCM	Content	Function	% by Weight
1E-3SPA	Anhydrous Na_2_SO_4_	Base salt	35.7
	Anhydrous borax	Nucleating agent	2.8
	NaCl	Melting point suppression	12.0
	SPA	Thickener	3.0
	Distilled water	Hydration	46.5
2E-3SPA	Anhydrous Na_2_SO_4_	Base salt	37.2
	Anhydrous borax	Nucleating agent	2.8
	NaCl	Melting point suppression	9.0
	SPA	Thickener	3.0
	Distilled water	Hydration	48.0

**Table 5:** Comparison of the derived properties of n-Hexadecane with literature values.

Parameter	Current Work T-History	Velez et al. [49] DSC
Latent heat: Solidification (kJ·kg^−1^)	234 ± 4.5	237
Solid-state specific heat (kJ·kg^−1^·K^−1^)	2.48 ± 0.04	-
Liquid-state specific heat (kJ·kg^−1^·K^−1^)	2.38 ± 0.01	2.22
Deviation in latent heat (%)	−1.2	
Deviation in liquid-state specific heat (%)	8	

**Table 6:** Melting and solidification temperature properties of 1E-3SPA for cycle 3.

Temperature (°C)	Sample 1	Sample 2	Sample 3	Average ± 95% Confidence Limit
Melting onset	21.3	21.4	21.2	21.3 ± 0.2
Melting end	26.7	25.5	26.4	26.2 ± 1.3
Supercooling	12.6	12.9	12.8	12.8 ± 0.3
Solidification	14.2	14.5	14.0	14.2 ± 0.5
Solidification end	9.5	9.3	9.6	9.4 ± 0.3
Temperature hysteresis	7.2	7.0	7.2	7.1 ± 0.3

**Table 7:** Properties of 1E-3SPA derived from enthalpy–temperature curves.

Thermal Property	Sample 1	Sample 2	Sample 3	Average ± 95% Confidence Limit
Latent heat: Melting (kJ·kg^−1^)	149.5	140.4	127.4	139.1 ± 22.5
Latent heat: Solidification (kJ·kg^−1^)	100.6	100.5	100.8	100.6 ± 0.3
Solid-phase specific heat (kJ·kg^−1^·K^−1^)	2.9	2.9	2.9	2.9 ± 0.1
Liquid-phase specific heat (kJ·kg^−1^·K^−1^)	3.3	3.4	3.4	3.4 ± 0.1
Enthalpy hysteresis (kJ·kg^−1^)	50.8	48.7	37	45.5 ± 15.1

**Table 8:** Temperature characteristics of 2E-3SPA derived from enthalpy–temperature curves.

Temperature (°C)	Sample 1	Sample 2	Sample 3	Average ± 95% Confidence Limit
Melting onset	21.5	21.4	21.3	21.4 ± 0.2
Melting end	28.0	28.2	27.5	27.9 ± 0.7
Supercooling	17.5	17.0	18.2	17.6 ± 1.3
Solidification	18.9	19.3	20.1	19.4 ± 1.3
Solidification end	9.7	9.2	9.8	9.6 ± 0.7
Temperature Hysteresis	2.6	2.2	1.2	2 ± 1.5

**Table 9:** Bulk properties of 2E-3SPA derived from enthalpy–temperature curves.

Thermal Property	Sample 1	Sample 2	Sample 3	Average ± 95% Confidence Limit
Latent heat: Melting (kJ·kg^−1^)	205.5	162.6	144.7	170.9 ± 63.4
Latent heat: Solidification (kJ·kg^−1^)	123.4	125.5	120.2	123.0 ± 5.4
Solid-phase specific heat (kJ·kg^−1^·K^−1^)	2.53	3.11	2.74	2.8 ± 0.6
Liquid-phase specific heat (kJ·kg^−1^·K^−1^)	3.5	3.5	3.4	3.5 ± 0.1
Enthalpy hysteresis (kJ·kg^−1^)	80	62.2	55.3	65.8 ± 25.8

**Table 10:** Probability density functions applied to input quantities for Monte Carlo simulations.

Input Quantities	Probability Density Function (PDF)	PDF Parameters
$T_{pcm}$ $,\;T_{ref}$ $\;\mathrm{and}\;T_{\infty }$	Normal	Mean = 0 °C, Standard deviation = 0.23 °C
$m_{pcm}$ $,\;m_{ref}$ $,m_{tube}$	Rectangular	±0.1 g
$C_{p,ref}$ $,C_{p,tube}$	Rectangular	±0.1 J·g^−1^·K^−1^

**Table 11:** Estimated values of bulk thermophysical properties of 2E-3SPA and their associated uncertainties.

Quantity	Estimated Value	Measurement Uncertainty
$C_{p,s}$ (kJ·kg^−1^·K^−1^)	2.6	±0.3
$C_{p,l}$ (kJ·kg^−1^·K^−1^)	3.4	
$\Delta H_{8-35{}^{\circ}C}$ (kJ·kg^−1^): Heating	241.74	±15
$\Delta H_{8-35{}^{\circ}C}$ (kJ·kg^−1^): Cooling	183.10	±13

**Table 12:** Thermophysical property comparison, 1E-3SPA vs. 2E-3SPA.

Property	1E-3SPA	2E-3SPA
Melting onset temperature (°C)	21.3 ± 0.2	21.4 ± 0.2
Melting end temperature (°C)	26.2 ± 1.3	27.9 ± 0.7
Degree of supercooling (°C)	1.6 ± 0.8	1.9 ± 2.6
Solidification temperature (°C)	14.2 ± 0.5	19.4 ± 1.3
Solidification end temperature (°C)	9.4 ± 0.3	9.6 ± 0.7
Temperature hysteresis (°C)	7.1 ± 0.3	2 ± 1.5
Latent heat of fusion (kJ·kg^−1^)	139 ± 22.5	171 ± 63.4
Latent heat of crystallization (kJ·kg^−1^)	101± 0.3	123 ± 5.4
Solid-phase specific heat (kJ·kg^−1^·K^−1^)	2.9 ± 0.1	2.8 ± 0.6
Liquid-phase specific heat (kJ·kg^−1^·K^−1^)	3.4 ± 0.1	3.5 ± 0.1
Vol. energy storage density (MJ·m^−3^)	230	282

**Table 13:** Volumetric energy densities of PCMs investigated in this study vs. commercially available PCMs.

PCM	Category	PCM Data Category	Volumetric Energy Storage Density (MJ·m^−3^)
1E-3SPA	Salt hydrate	Current study	230
2E-3SPA			282
RT25HC [53]	Paraffin	Commercial	204
SP25E2 [53]	Salt hydrate		288
Climsel C24 [54]	Salt hydrate		176

## Data Availability

The raw data supporting the conclusions of this article will be made available by the authors on request.

## Nomenclature

a	Proportionality constant in the power law curve fit
$A_{o}$	Outer surface area of the test tube (m^2^)
b	Power law coefficient (−)
Bi	Biot number
c	Offset in the power law curve fit (W)
$C_{p}$	Specific heat (kJ·kg^−1^·K^−1^)
dt	Elemental change in time (second)
dT	Elemental change in temperature (°C)
h	Convective heat transfer coefficient (W·m^−2^·K^−1^)
ΔH	Step enthalpy change (kJ·kg^−1^)
H	Enthalpy (kJ·kg^−1^)
$H_{f}$	Latent heat of fusion (kJ·kg^−1^)
$H_{0}$	Reference enthalpy (kJ·kg^−1^)
I	Area under the temperature–time curve (°C-sec)
k	Thermal conductivity (W·m^−1^·K^−1^)
m	Mass (gram)
N	Number of PCM intervals
q	Heat flow rate (W)
$R_{i}$	Inner radius of the test tube (mm)
$R_{o}$	Outer radius of the test tube (mm)
$R_{th}$	Thermal resistance (°C·W^−1^)
t	Time (second)
Δt	Duration of the interval (second)
T	Temperature (°C)
$\bar{T}$	Average temperature (°C)
$T_{m}$	Melting temperature (°C)
$T_{s}$	Solidification temperature (°C)
$T_{SC}$	Supercooling temperature (°C)
ΔT	Step change in temperature (°C)
$\Delta T_{int}$	Minimum allowed temperature interval (°C)
**Greek Symbols:**	
ε	Standard uncertainty in temperature measurement (°C)
**Subscripts:**	
∞	Ambient
avg	Average
cond	Conduction
conv	Convection
int	Interval
l	Liquid
m	Melting point
pcm	Phase change material
ref	Reference
s	Solid
sen	Sensor
tube	Test tube
**Abbreviations:**	
CFD	Computational fluid dynamics
CMC	Carboxymethyl cellulose
DOE	Department of Energy
DSC	Digital scanning calorimetry
FEA	Finite element analysis
HVAC	Heating, ventilation, and air conditioning
IQR	Interquartile range
JCGM	Joint Committee for Guides in Metrology
PAM	Polyacrylamide
PCM	Phase change material
PDF	Probability density function
SAT	Sodium acetate trihydrate
SLM	Shape language modeling
SPA	Sodium polyacrylate
SSD	Sodium sulfate decahydrate
TES	Thermal energy storage
